# RETRACTED: Liu et al. Antiproliferative Activity of Triterpene Glycoside Nutrient from Monk Fruit in Colorectal Cancer and Throat Cancer. *Nutrients* 2016, *8*, 360

**DOI:** 10.3390/nu16172846

**Published:** 2024-08-26

**Authors:** Can Liu, Longhai Dai, Yueping Liu, Long Rong, Dequan Dou, Yuanxia Sun, Lanqing Ma

**Affiliations:** 1 Key Laboratory of Urban Agriculture (North) of Ministry of Agriculture, Beijing University of Agriculture, Beijing 102206, China; liucan808@163.com; 2 National Engineering Laboratory for Industrial Enzymes, Tianjin Institute of Industrial Biotechnology, Chinese Academy of Sciences, Tianjin 300308, China; dai_lh@tib.cas.cn (L.D.); sun_yx@tib.cas.cn (Y.S.); 3 Beijing Collaborative Innovation Center for Eco-Environmental Improvement with Forestry and Fruit Trees, Beijing 102206, China; 4 School of Biological Science and Medical Engineering, Beihang University, Beijing 100191, China; ronglong64@163.com

The journal retracts the article, “Antiproliferative Activity of Triterpene Glycoside Nutrient from Monk Fruit in Colorectal Cancer and Throat Cancer” [1], cited above.

Following publication, the authors contacted the publisher to raise concerns regarding the reliability of a number of the images presented in this article [1].

Adhering to our standard procedure, an investigation was conducted by the Editorial Office and Editorial Board that confirmed the presence of a partial figure duplication within this publication [1] and a number of panels duplicated from an earlier publication [2], produced by a different author group.

As a result, the Editorial Board and the authors have agreed that the findings of this article could not be relied upon and have decided to retract this publication [1], as per MDPI’s retraction policy (https://www.mdpi.com/ethics#_bookmark30, accessed on 15 August 2024).

This retraction was approved by the Editor-in-Chief of the journal *Nutrients*.

The authors have agreed to this retraction.
